# Vaccine mRNA Can Be Detected in Blood at 15 Days Post-Vaccination

**DOI:** 10.3390/biomedicines10071538

**Published:** 2022-06-28

**Authors:** Tudor Emanuel Fertig, Leona Chitoiu, Daciana Silvia Marta, Victor-Stefan Ionescu, Valeriu Bogdan Cismasiu, Eugen Radu, Giulia Angheluta, Maria Dobre, Ana Serbanescu, Mihail Eugen Hinescu, Mihaela Gherghiceanu

**Affiliations:** 1 Victor Babeș National Institute of Pathology, 050096 Bucharest, Romania; leona.chitoiu@ivb.ro (L.C.); daciana.marta@ivb.ro (D.S.M.); victor.ionescu@ivb.ro (V.-S.I.); vcismasiu@gmail.com (V.B.C.); maria.dobre@ivb.ro (M.D.); mhinescu@yahoo.com (M.E.H.); mgherghiceanu@yahoo.com (M.G.); 2 Faculty of Medicine, Carol Davila University of Medicine and Pharmacy, 050474 Bucharest, Romania; eugen.radu@umfcd.ro (E.R.); giulia.angheluta@stud.umfcd.ro (G.A.); 3 Faculty of Medicine, Titu Maiorescu University, 040441 Bucharest, Romania; 4 Laboratory of Molecular Biology and Pathology Research, Emergency University Hospital Bucharest, 050098 Bucharest, Romania; 5 Cantacuzino NIRDMM-Research and Development Center, 050096 Bucharest, Romania; ana.serbanescu92@gmail.com

**Keywords:** COVID-19, mRNA vaccine, lipid nanoparticles, biodistribution, spike protein

## Abstract

COVID-19 mRNA vaccines effectively reduce incidence of severe disease, hospitalisation and death. The biodistribution and pharmacokinetics of the mRNA-containing lipid nanoparticles (LNPs) in these vaccines are unknown in humans. In this study, we used qPCR to track circulating mRNA in blood at different time-points after BNT162b2 vaccination in a small cohort of healthy individuals. We found that vaccine-associated synthetic mRNA persists in systemic circulation for at least 2 weeks. Furthermore, we used transmission electron microscopy (TEM) to investigate SARS-CoV-2 spike protein expression in human leukemic cells and in primary mononuclear blood cells treated in vitro with the BNT162b2 vaccine. TEM revealed morphological changes suggestive of LNP uptake, but only a small fraction of K562 leukemic cells presented spike-like structures at the cell surface, suggesting reduced levels of expression for these specific phenotypes.

## 1. Introduction

COVID-19 vaccines using messenger RNA (mRNA) technology have been developed at unprecedented speed to combat the global spread of SARS-CoV-2 and to date remain safe and effective at preventing severe disease, hospitalisation and death. In the US alone, it has been estimated that COVID-19 vaccines averted almost one-quarter of a million deaths [1]. Importantly, advances in mRNA technology likely signal the start of a revolution in medicine, whereby such molecules could help prevent other infectious diseases or be used in the treatment of cancer [2].

Briefly, the two licensed COVID-19 mRNA vaccines contain synthetic mRNA that codes the proline-stabilised prefusion conformation of the SARS-CoV-2 spike glycoprotein (S-protein) [3,4]. The receptor-binding domain (RBD) of the S-protein represents the main target for neutralising antibodies [5]. The mRNA is packaged into lipid nanoparticles (LNPs), which are then delivered to cells where the S-protein is expressed and processed for immune recognition. The biodistribution and persistence of LNP-mRNA vaccine formulations, for COVID-19 and other diseases, have been studied in rodents and non-human primates. Intramuscular injection leads to an initial accumulation at the injection site, after which LNPs are rapidly transported to proximal lymph nodes (LNs) by passive draining as well as actively carried by professional antigen-presenting cells and neutrophils [6,7]. From here, the remaining unprocessed vaccine particles reach systemic circulation and, depending on the composition of the lipid shell, may be targeted to the liver, spleen or other organs [2]. Data generated in rat models, submitted to the European Medicines Agency (EMA) by Pfizer-BioNTech and Moderna, indicate that proprietary LNP formulations would reach most tissues, but they preferentially concentrate at the injection site and liver in the case of BNT162b2 [8] and injection site, LNs and spleen in the case of mRNA-1273 [9].

Some LNPs formulations degrade quickly within tissues [10]; however, proprietary ionisable lipids in BNT162b2 (ALC-0315) were expected to be completely eliminated in rat livers after as much as 6 weeks [8]. Protein expression was shown to persist 7 to 10 days in previous studies on mice [10,11] and at least 9 days in BNT162b2 studies submitted to EMA [8].

A recent ultrasensitive single-molecule assay was however able to detect the S-protein in the plasma of some mRNA-1273 COVID-19 vaccinees at 15 days following injection [12], while in another study, both mRNA and S-protein could be found in axillary LNs after 60 days [13]. Moreover, extracellular vesicles decorated with S-proteins persist up to 4 months after vaccination with BNT162b2 [14]. This raises the possibility that LNP–mRNA complexes remain in circulation for extended periods of time, retaining their ability to induce S-protein expression in contacted cells.

The biodistribution and functional half-life of vaccine-associated S-protein mRNA in humans are currently unknown. In this study, we investigated the persistence of the BNT162b2 COVID-19 mRNA vaccine in the blood of vaccinated individuals, and we tested its ability to induce S-protein expression in cultured human white blood cells (WBCs). We believe that any human biodistribution data are essential to our understanding of immune responses following COVID-19 vaccination and can serve the development of future mRNA vaccines.

## 2. Materials and Methods

### 2.1. Blood Collection and RNA Extraction

Healthy individuals scheduled to be vaccinated with the Comirnaty COVID-19 mRNA vaccine (Pfizer BioNTech, New York, NY, USA) and unvaccinated controls were invited to participate in this study. Blood was collected after written and informed consent, in accordance with the rules of the Declaration of Helsinki of 1975 and following approval from the ethics committee at Victor Babeș National Institute of Pathology, Bucharest, Romania.

Whole blood (500 μL) was collected in EDTA tubes and centrifuged (500× *g*, 10 min, 4 °C) for plasma separation. Anti-spike (S) and anti-nucleocapsid (N) IgG in serum were measured using a COVID-19 ELISA diagnostic kit (G1032, Vircell Microbiologists, Granada, Spain). As per manufacturer instructions, index values above 6 were considered positive for IgG antibodies.

Total RNA was extracted from 200 μL plasma using the miRNeasy kit (217184, Qiagen, Hilden, Germany), following the manufacturer’s protocol. Total RNA was also extracted from 300 μL of WBCs using the PureLink Total RNA Blood Purification kit (K1560-01, Invitrogen, Carlsbad, CA, USA), following the manufacturer’s protocol. The purity and concentration of samples were determined spectrophotometrically, using a Nanodrop 2000 (Thermo Fisher Scientific, Waltham, MA, USA). OD_260/280_ varied from 1.33 to 1.52 for plasma and 2.02 to 2.1 for WBCs.

### 2.2. Reverse Transcription and qPCR

RNA extracted from WBCs and plasma was treated with DNase I (79254, Qiagen, Hilden, Germany), and a volume of 2.5 µL was reverse transcribed using the GoScript™ ReverseTranscription System (A5000, Promega, Madison, WI, USA) according to the manufacturer’s instructions. Reactions used both random and oligo(dT_15_) primers, had a final reaction volume of 10 µL, and included no-RT controls.

Two custom candidate primer pairs were designed for the qPCR quantification of BNT162b2 and purchased from Eurogentec (Seraing, Belgium). Primers were designed to be specific to the publicly available, codon-optimised vaccine mRNA sequence [15] but not to viral RNA encoding the S-protein. Primers had no BLAST hits for coronavirus and human genome or transcript sequences, and they had a predicted melting temperature of at least 63 °C. One pair was selected based on results of PCR assays performed with known negative samples (WBC gDNA, WBC cDNA, plasma cDNA and total RNA from unvaccinated individuals), as well as with the BNT162b2 vaccine mRNA and a custom synthetic single-stranded DNA (ssDNA) positive control. The ssDNA was designed to correspond to cDNA generated by reverse transcription of vaccine mRNA and was purchased from Eurogentec (Seraing, Belgium). Importantly, the selected primer pair failed to amplify two commercially available SARS-CoV-2 S-gene positive controls (Exact Diagnostics SARS-CoV-2 Standard, COV019, Bio-Rad, Hercules, CA, USA and AcroMetrix Coronavirus 2019 (COVID-19) RNA Control (RUO), 954519, Thermo Fisher Scientific, Waltham, MA, USA), thus confirming the absence of specific hybridisation to viral nucleic acids from potentially infected individuals. The sequences of selected primers were 5′-GTGGATCTGCCCATCGGCATC (forward) and 5′-GTCCATCCGCTGCTGCTATCG (reverse).

All qPCR reactions were performed using GoTaq^®^ qPCR Master Mix (A6001, Promega, Madison, WI) with BRYT Green^®^ Dye at a final volume of 10 µL. Assays included 1 µL of reverse transcription product or water for negative control, and forward and reverse primers at a final concentration of 200 nM. After an initial enzyme activation step of 10 min at 95 °C, 40 cycles of amplification were performed (15 s at 95 °C, 1 min at 65 °C) and a final step of heating from 60 to 95 °C with 5 fluorescence readings per °C for melt curve analysis. Each qPCR experiment included a series of 10-fold dilutions of the ssDNA positive control from 10 to 10^7^ copies/reaction, which were calculated based on the total quantity indicated by the manufacturer (42.2 nmol, Eurogentec, Seraing, Belgium). An LC480 II real-time PCR system running LightCycler^®^ 480 software version 1.5 (Roche Diagnostics, Mannheim, Germany) was used for data acquisition and initial analysis. The number of template copies per reaction was calculated for each experiment using the linear regression of known ssDNA concentrations on Cq values ($10^{\left(Cq-intercept\right)/slope}$) [16]. The amount of input RNA for each qPCR reaction was determined using the dilution factor of the extracted RNA across DNase I treatment and reverse transcription. The normalised mRNA copy number was calculated by dividing the mRNA copy number to the amount of input RNA. The limit of quantification (LoQ) was defined as the lowest standard with a coefficient of variation of less than 35% [17]. Here, the LoQ was determined to be 10 template copies per reaction, as the corresponding standard was detected with a 5.26% coefficient of variation (calculated as 100 × standard deviation/mean of Cq values, [17]). The calculated assay efficiency was between 1.92 and 2.14 ($10^{-1/slope}-1$, [18]), standard curves showed R^2^ correlation coefficients between 0.991 and 0.999, and the melt curve peak for the specific product was calculated at 86.78–86.96 °C.

Data analysis was performed in R version 3.6.3 [19]. Graphs were generated with the ggplot2 package (version 3.3.3) [20].

### 2.3. Cell Culture

Vials of frozen COVID-19 mRNA Vaccine (Comirnaty/BNT162b2, Pfizer BioNTech, New York, NY, USA) were kindly provided by the Romanian Ministry of Health. All vaccine dilutions were performed using sterile 0.9% sodium chloride. Unless otherwise stated, all cell culture reagents and consumables were purchased from Thermo Fisher Scientific (Waltham, MA, USA).

Human granulocytic blasts (K562, CCL-243™, ATCC, Manassas, VA, USA) and human promyeloblasts (HL-60, ECACC, 98070106, Merck KGaA, Darmstadt, Germany) were cultured at 37 °C with 5% CO_2_, in RPMI-1640 supplemented with 10% FBS and 1% penicillin–streptomycin. Cells were seeded in 12-well plates at 5 × 10^5^ cells per well and in medium without antibiotics; then, they were incubated with 10 μg total vaccine mRNA per well, for 3, 12 or 24 h. Control cells received PBS.

Peripheral blood mononuclear cells and platelets (PBMCp) were isolated from two healthy, unvaccinated volunteers (both male, aged 21) without a history of SARS-CoV-2 infection. Fresh blood (4 mL) was collected after informed consent, carefully layered onto Histopaque^®^-1077 medium (Sigma-Aldrich, Burlington, MA, USA) at equal volume, and then, the tubes were centrifuged at 400× *g* for 40 min. The top-most layer (plasma) was discarded, whereas the mononuclear cell layer and platelets were carefully picked and transferred to a sterile centrifuge tube. Cells and platelets were then washed twice with PBS, resuspended in DMEM supplemented with 10% FBS (without antibiotic), and seeded in 24-well plates at 1 × 10^6^ cells/well. Cells were then incubated with 10 μg total vaccine mRNA per well, for 3, 12 or 24 h. PBS was added to control wells.

### 2.4. Transmission Electron Microscopy

For room-temperature transmission electron microscopy (TEM), cells were fixed for 2 h with a pre-warmed solution comprising 2.5% glutaraldehyde and 1.4% sucrose (pH 7.2); then, they were processed for epoxy resin embedding, as detailed by the manufacturer (Agar100 Resin Kit, Agar Scientific, Essex, UK) and as described in previous work from this group [21]. Sectioning was performed on a Leica EM UC7 ultramicrotome (Leica, Wetzlar, Germany), and the ultra-thin sections (40–60 nm) were mounted on carbon and Formvar-coated 50-mesh copper grids and counterstained with 1% uranyl acetate and Reynolds lead citrate (Agar Scientific, Essex, UK). Imaging was done using a 200 kV Talos F200C TEM, equipped with a 4K × 4K Ceta camera (Thermo Fisher Scientific, Waltham, MA, USA).

### 2.5. Western Blot

Samples treated with vaccine-associated mRNA, and untreated controls were lysed with Laemmli loading buffer and boiled for 10 min at 95 °C; then, they were loaded and separated by SDS-PAGE on a 10% gel. Recombinant SARS-CoV-2 RBD was used as a positive control and loaded at increasing concentrations from 0.25 to 4 pmol (6.25 to 100 ng total protein). The RBD was obtained by transfection of Expi293F™ cells (A14527, Thermo Fisher Scientific, Waltham, MA, USA) with pcDNA3-SARS-2-RBD-8his (145145, Addgene, Watertown, MA, USA) and was a kind gift from Dr. Costin-Ioan Popescu at the Viral Glycoprotein Department, Biochemistry Institute of the Romanian Academy of Science, 060031 Bucharest, Romania). 

The gel was transferred onto a PVDF membrane and blocked with a 5% blocking buffer (milk) at 4 °C overnight. The membrane was blotted with 1:250 anti-SARS-CoV-2 spike protein (RBD) rabbit polyclonal antibody (PA5-114529, Thermo Fisher Scientific, Waltham, MA, USA) for 2 h at room temperature, which was followed by a 1 h incubation with 1:40,000 goat anti-rabbit IgG secondary antibody coupled with horseradish peroxidase (HRP, A9169, Sigma-Aldrich, Burlington, MA, USA) and visualised using SuperSignal West Pico PLUS Chemiluminescent Substrate (34577, Thermo Fisher Scientific, Waltham, MA, USA).

## 3. Results

### 3.1. Study Participants

Between January and May 2021, we collected blood samples from 16 individuals who had recently received the first or second dose of the BNT162b2 vaccine (Table 1). Of these, 11 were female (68.75%) and five were male (31.25%), with a median age of 31 years (range 21 to 50 years old). All participants were healthy, without known comorbidities and declared no history of SARS-CoV-2 infection. None of the participants reported significant adverse effects following vaccination (Table S1). Blood was collected only once for most volunteers (*n =* 10) and twice or three times for others (*n =* 6), at various intervals, covering the range from 1 to 27 days following vaccination and totalling 23 samples. We also included three healthy volunteers (two males and one female, median age 46 years) without a history of SARS-CoV-2 infection and who had not taken the vaccine, as verified by anti-N and anti-S IgG status (Table S2).

Additionally, in December 2021, we enrolled a healthy, 49-year-old female volunteer (B3), from whom we collected blood seven times, at regular intervals, starting with the day of the third (booster) shot of BNT162b2 and ending at day 14 (Table 1).

### 3.2. Vaccine mRNA Remains in Circulation for at Least 15 Days

We used qPCR to investigate the presence and persistence of synthetic mRNA in blood after one, two and three doses of BNT162b2, assuming that the biodistribution of the vaccine is similar after each dose. We also aimed to probe whether vaccine mRNA was associated with circulating WBCs, either as a result of cell–LNP interactions at the injection site and in draining LNs or of random collisions in circulation. Consequently, we separated the plasma and cellular fractions and analysed them independently for all individuals.

**Figure 1:**
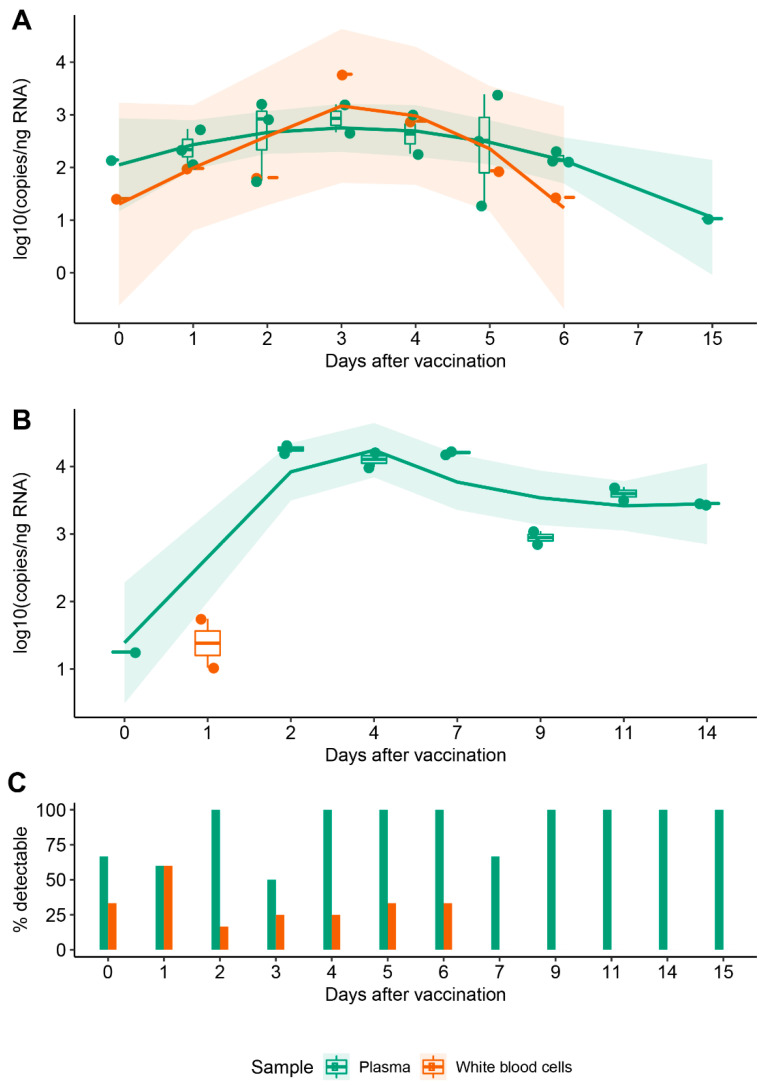
Persistence of synthetic mRNA in blood following vaccination. (**A**) Distribution of synthetic mRNA copy number normalised to the equivalent quantity of input RNA in a cohort of 16 individuals; each point represents an independent biological sample. (**B**) Distribution of synthetic mRNA copy number normalised to the equivalent quantity of input RNA in a single individual; technical replicates are shown for all timepoints analyzed. For (**A**,**B**), trendlines were fitted using locally estimated scatterplot smoothing (LOESS) regression to observe variations of mRNA in plasma (green) and cellular fraction (orange) in the days following vaccination. Only data corresponding to copy numbers above LoQ = 10 are shown. (**C**) Cumulated likelihood of detecting synthetic mRNA in plasma and cellular fractions across subjects and days following vaccination.

Template copy numbers decreased with the day of sampling. In plasma, mRNA was immediately detectable at just hours following vaccination, remained detectable when sampled at 6 and 15 days (Figure 1A, green), but was below the limit of quantification (LoQ) for one sample at 27 days. Samples from negative controls did not amplify. For subject B3, we observed a similar trend for plasma: vaccine-associated mRNA became detectable immediately after vaccination and remained significantly above the LoQ at day 14 (Figure 1B, green). Interestingly, vaccine mRNA was detected in the cellular fraction up to day 6 in some samples from our cohort, whereas for B3, it was only detectable at one day after vaccination (Figure 1A,B, orange). It has to be noted that the likelihood of detecting vaccine mRNA in the cellular fraction decreased significantly at 24 h from injection, which was in contrast to plasma in which mRNA remained consistently detectable up to day 15 (Figure 1C).

### 3.3. WBCs Are Unlikely Candidates for S-Protein Expression In Vitro

We next asked whether the presence of vaccine mRNA in the WBC fraction in the first days following injection was due to uptake of vaccine LNPs and if this leads to expression of the S-protein in these cells. To test this, we exposed cultured human myeloid cells (K562 and HL-60) and isolated peripheral blood mononuclear cells and platelets (PBMCp) to the BNT162b2 vaccine. We used TEM to identify suggestive morphological changes in these cells and the potential expression of S-protein.

**Figure 2:**
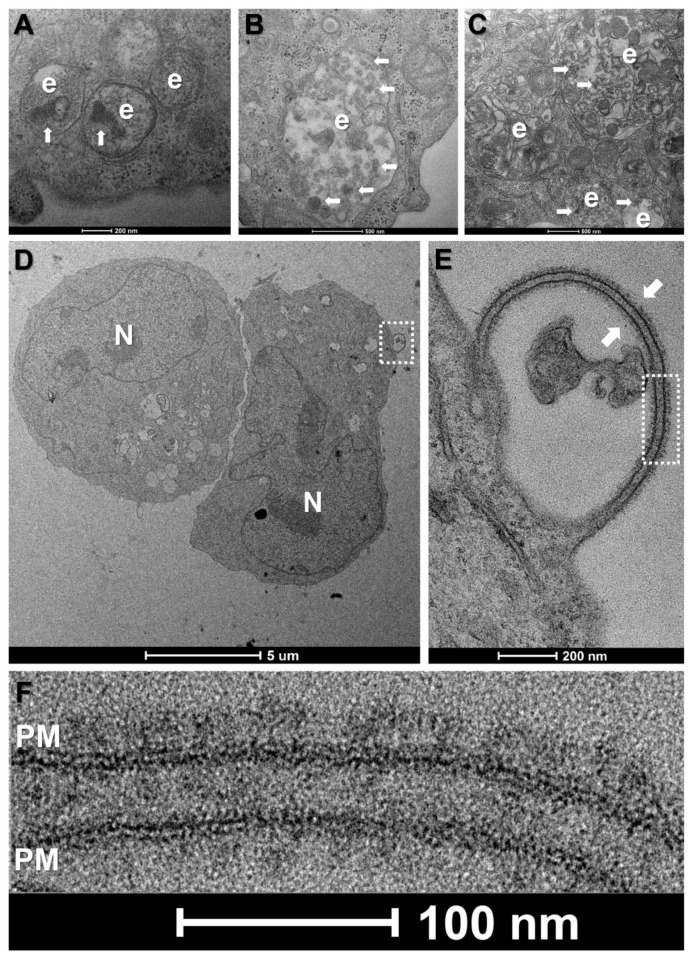
Morphological changes in primary and leukemic blood cells at 12 h incubation with the mRNA vaccine. (**A**–**C**) Enlarged endolysosomal compartments (**e**) in HL-60 cells (**A**), K562 cells (**B**) and primary human mononuclear phagocytes (**C**), containing electron dense particles (white arrows), indicative of LNP uptake. (**D**) Low-magnification view of two K562 cells. *N-nucleus.* (**E**) Higher magnification view of boxed area in (**D**) showing a membrane protrusion decorated on both sides with spike-like structures. (**F**) Higher magnification view of boxed area in (**E**), showing typical morphology and size of full-length, prefusion SARS-CoV-2 spike proteins, clustering on the lipid bilayer of the plasma membrane (PM).

In our hands, despite very high vaccine concentrations (10 μg mRNA per 1 × 10^6^ cells), morphological changes were subtle for all tested cell types. Common features for vaccine-treated cells at all time-points were enlarged trans-Golgi cisternae, ERGIC vesicles and rough endoplasmic reticulum tubules as well as more prominent endosomes and lysosomes (data not shown). Notably, the endolysosomal compartments of vaccine-treated HL-60, K562 and primary mononuclear phagocytes were enriched with electron dense, multi-layered lipid structures as compared to controls, suggesting the successful endocytosis of LNPs (Figure 2A–C).

In the case of K562 cells, we observed spike-like structures clustering on isolated protrusions of the plasma membrane at 12 h incubation with the vaccine (Figure 2D). These structures were tightly packed, approximately 20 nm in length and presented the typical morphology of full-length, prefusion SARS-CoV-2 S-proteins, with a narrow stalk and a wider head region (Figure 2E,F). However, of approximately 100 cells analysed, only three presented spikes, suggesting low levels of expression. Such spikes were absent in K562 cells at 3 and 24 h incubation and were also absent in PBS-treated controls. By contrast, spike-like structures were conspicuously absent on vaccine-treated HL-60 cells or at the cell surface of the lymphocytes, mononuclear phagocytes and platelets identified in the PBMCp fraction, regardless of incubation times.

**Figure 3:**
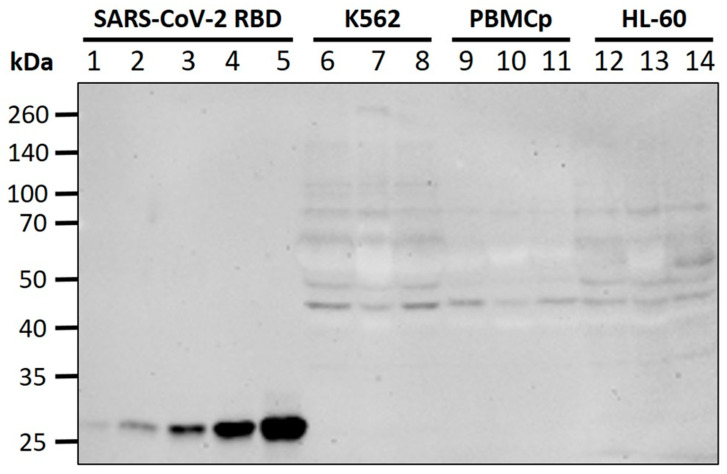
Western blot for S-protein expression. Lanes 1–5: recombinant SARS-CoV-2 S-protein RBD (0.25 pmol, 0.5 pmol, 1 pmol, 2 pmol, 4 pmol); Lanes 6–8: K562 lysates (control, 12 h, 3 h); Lanes 9–11: PBMCp lysates (control, 24 h, 3 h); Lanes 12–14: HL-60 lysates (control, 12 h, 3 h). No difference in antibody binding was seen between vaccine-treated cells and negative controls at the expected molecular weights for full-length S-protein or fragments.

To verify whether the absence of visible full length S-protein at the cell surface in TEM is due to it being retained or partially degraded in intracellular compartments, we performed Western blot on all cell lines and at all time-points, using an antibody against the SARS-CoV-2 RBD. No specific bands were detected in the region of 180 kDa (full-length S-protein) or 110 kDa (S1 fragment), including for K562 cells. The antibody successfully detected a recombinant SARS-CoV-2 RBD (25 kDa region of the S1 fragment) to a lower limit of 0.25 pmol (Figure 3). Overall, these data confirm S-protein expression levels below our limit of detection for the tested cell lines.

## 4. Discussion

The biodistribution and pharmacokinetics of LNP-mRNA particles of the current generation of COVID-19 mRNA vaccines are of great interest, as they directly impact the immunogenicity and potential toxicity of these platforms. Although similar formulations were tracked in various tissues of rodents up to 5 days post injection by Moderna [9] and 14 days by Pfizer-BioNTech [8], no human biodistribution data are currently available for any of the two licensed products. In this study, we investigated the persistence of mRNA in circulation following vaccination with BNT162b2 and the susceptibility of leukemic and primary blood cells for LNP-mRNA uptake and S-protein expression.

We first used qPCR on blood samples collected from recently vaccinated volunteers and showed that vaccine-associated synthetic mRNA persists in circulation at least 2 weeks following injection with either dose of BNT162b2, albeit decreasing after 4 days. Our results show extended plasma clearance times compared to estimates presented by mRNA vaccine manufacturers. Data provided by Pfizer-BioNTech showed luciferase-encoding RNA constructs containing proprietary ionisable lipids were terminally cleared in the plasma of rats after a maximum of 6 days [8]. Similarly, Moderna LNP-mRNA constructs became undetectable in the plasma of rats within one day, as measured by a multiplex branched DNA assay [9].

We detected vaccine mRNA overwhelmingly in the plasma fraction, suggesting it mostly circulates freely and not actively transported by WBCs. Moreover, it was likely circulating in its lipid-encapsulated form, as naked mRNA would have been rapidly degraded in the extracellular environment [22]. We therefore expect these particles to also retain their ability to induce S-protein expression in susceptible cells over this time period, and we infer that such low-level, prolonged antigenic stimulation may contribute to effective immune responses, especially the proliferation of memory T-cells [23].

This analysis has a number of limitations. First, despite relative demographic homogeneity, our study cohort was small, and individual variations relating to the clearance rate of vaccine particles at the injection site may have contributed to differences in circulating vaccine mRNA between subjects and days. Second, we did not perform WBC counts for samples used for RNA extraction which, coupled with the fact that the total RNA load of WBCs is known to differ between subjects in both quantity and expression patterns, may have introduced errors in our quantification of vaccine mRNA in the cellular fraction.

In this study, we also investigated potential S-protein expression as a result of interactions of vaccine LNP–mRNA particles with human WBC phenotypes and with platelets. We chose the leukemic cell lines HL-60 and K562, as these are of myeloid lineage and have successfully been used for lipofection studies, including of RNA species [24]. Additionally, in an attempt to replicate potential interactions between circulating cells and LNP–mRNA in human blood, we treated PBMC and platelets collected from healthy, unvaccinated donors with the vaccine. The PBMC fraction includes diverse lymphocyte populations and monocytes, which both have been shown to be efficient at LNP uptake and mRNA translation [7].

To our surprise, despite the exposure of these varied cell types to high vaccine concentrations, TEM only revealed S-protein-like structures on the membrane surface of a small fraction of K562 cells and only at 12 h of incubation with BNT162b2. Interestingly, the putative S-proteins were restricted to bulging regions of the plasma membrane; however, the significance of these membrane extensions is unknown and warrants further study.

Notably, our TEM analysis of S-protein expression in cultured cells was dependent on the inherent limitations of plastic sectioning, whereby full-length S-proteins decorating the cell surface are easily recognisable only when sectioning is done parallel to the long axis of the molecule. Moreover, some expressed S-proteins would be broken down into fragments by proteasomes, and then, only these fragments coupled with major histocompatibility complex molecules to be displayed at the membrane surface [2]. To verify whether these factors contributed to the rarity of full-length S-proteins in TEM, we performed Western blotting with an antibody directed against the S-protein RBD; however, no specific band was detected for either cell line or incubation time.

We suggest that the tandem of methods used herein indicates inefficient mRNA escape from endosomal compartments for these cell types and using these experimental conditions but not inefficient LNP uptake. TEM of vaccine-treated K562 and HL-60 cells and of primary mononuclear phagocytes revealed frequent large endolysosomes containing numerous electron-dense multi-layered structures, which was suggestive of accumulated LNPs. This apparent discrepancy may be explained by data from a recent study showing that the enhanced retention of LNP–mRNA particles in early endosomes can lead to acidification defects and arrest in endosomal maturation, which impedes mRNA escape [25].

In conclusion, we showed that BNT162b2 vaccine mRNA remains in the systemic circulation of vaccinated individuals for at least 2 weeks, during which it likely retains its ability to induce S-protein expression in susceptible cells and tissues. We also showed that WBCs and platelets are not favourable targets for S-protein expression in vitro. More complex human biodistribution and pharmacokinetics studies are required to elucidate the tissue tropism of mRNA vaccine particles as well as uptake and transfection efficiencies for other cell types. This would serve to further optimise future mRNA vaccine formulations.

## Figures and Tables

**Table 1:** Characteristics of enrolled subjects. Each letter of the participant ID designates an individual, while numbers represent dose and day of sampling, respectively.

Participant ID	Age (Years)	Sex	Vaccine	Doses Received	Time (Days)			
					Between Doses	Blood Collection after 1st Dose	Blood Collection after 2nd Dose	Blood Collection after 3rd Dose
A1.1	37	M	BNT162b2	1		1		
B1.1	49	F	BNT162b2	1		1		
C1.2	37	M	BNT162b2	1		2		
D1.3	21	F	BNT162b2	1		3		
E1.3	21	M	BNT162b2	1		3		
F1.4	21	F	BNT162b2	1		4		
G1.4	21	M	BNT162b2	1		4		
H1.5	33	F	BNT162b2	1		5		
I1.6	21	F	BNT162b2	1		6		
G1.6	21	M	BNT162b2	1		6		
H2.0	33	F	BNT162b2	2	21		0	
J2.1	37	F	BNT162b2	2	21		1	
B2.2	49	F	BNT162b2	2	21		2	
K2.2	50	F	BNT162b2	2	21		2	
J2.2	37	F	BNT162b2	2	21		2	
C2.3	37	M	BNT162b2	2	21		3	
L2.3	37	M	BNT162b2	2	21		3	
M2.5	25	F	BNT162b2	2	21		5	
N2.5	34	F	BNT162b2	2	21		5	
O2.6	35	F	BNT162b2	2	21		6	
P2.7	21	F	BNT162b2	2	21		7	
B2.15	49	F	BNT162b2	2	21		15	
M2.27	25	F	BNT162b2	2	21		27	
CTRL1	31	M						
CTRL2	68	F						
CTRL3	39	M						
B3	49	F	BNT162b2	3	21/272			0, 1, 2, 4, 7, 9, 11, 14

## Data Availability

More detailed records of the subjects included in this study cannot be made available due to data-sharing restrictions. Additional results have been included in the Supplemental Materials section. Transmission electron microscopy data can be provided upon reasonable request.

## Supplementary Materials

The following supporting information can be downloaded at: https://www.mdpi.com/article/10.3390/biomedicines10071538/s1, Table S1: Prevalence of side effects in participants after the first and second dose of BNT162b2 mRNA COVID-19 vaccine; Table S2: Levels of anti-spike and anti-nucleocapsid SARS-CoV-2 IgG in the serum of individuals enrolled in the study.
