# Supplementary material for: Vaccine mRNA Can Be Detected in Blood at 15 Days Post-Vaccination
Source: Biomedicines. 2022 Jun 28;10(7):1538. doi: 10.3390/biomedicines10071538 (PMC9313234; doi:10.3390/biomedicines10071538)
Supplement: Supplementary file 1 [file biomedicines-10-01538-s001.zip › biomedicines-1765117-supplementary.pdf]

Supplemental Data:

Side Effects	Number of participants (%)
Pain at injection site	11 (68.7)
Headache	6 (37.5)
Fever and/or chills	5 (31.2)
Fatigue	3 (18.7)
Arthralgia	3 (18.7)
Myalgia	3 (18.7)
Lymphadenopathy	3 (18.7)
Nausea	1 (6.2)
Tachycardia	1 (6.2)
Hypertension	1 (6.2)
Allergic reaction	0

Table S1: Prevalence of side effects in participants after the first and second dose of BNT162b2 mRNA COVID-19 vaccine.

Participant ID	Antibody levels (index)
**A1.1**	3.9
**B1.1**	3
**C1.2**	n/a
**D1.3**	2.6
**E1.3**	n/a
**F1.4**	4.7
**G1.4**	2.3
**H1.5**	3.5
**I1.6**	3.3
**G1.6**	3.8
**H2.0**	n/a
**J2.1**	n/a
**B2.2**	51.1
**K2.2**	80.6
**J2.2**	4.8
**C2.3**	18
**L2.3**	7.9
**M2.5**	22.8
**N2.5**	33.8
**O2.6**	48.8
**P2.7**	88.6
**B2.15**	40.6
**M2.27**	n/a
**CTRL1**	3.7
**CTRL2**	4.8
**CTRL3**	4.2
**B3 before booster**	5.4
**B3 after booster – days 2, 4, 7, 9, 11, 14**	8.9, 10.5, 154.7, 164.1, 153.1, 153.4

Table S2: Levels of anti-spike and anti-nucleocapsid SARS-CoV-2 IgG in the serum of individuals enrolled in the study. Levels are expressed as indices, with values above 6 considered positive. Each letter of the participant ID designates an individual, while numbers represent vaccine dose and day of sampling, respectively. CTRL – negative control, n/a – not analyzed.
